# Giant left pheochromocytoma with vascular anomalies and pelvic horseshoe kidney: a case report

**DOI:** 10.1186/s12894-023-01370-y

**Published:** 2023-12-08

**Authors:** Emily Biben, Lakshmi Menon, Neriman Gokden, Matthew F. Spond, Joshua M. Eichhorn, Ahmet Murat Aydin

**Affiliations:** 1 Department of Urology, University of Arkansas for Medical Sciences, 4301 W Markham St, Little Rock, AR 72205 USA; 2 Department of Endocrinology, University of Arkansas for Medical Sciences, Little Rock, AR USA; 3 Department of Pathology, University of Arkansas for Medical Sciences, Little Rock, AR USA; 4 Department of Anesthesiology, University of Arkansas for Medical Sciences, Little Rock, AR USA; 5 Department of Radiology and Nuclear Medicine, University of Arkansas for Medical Sciences, Little Rock, AR USA

**Keywords:** Adrenal gland Neoplasm, Adrenalectomy, Horseshoe kidney, Left retro-aortic renal vein, Pheochromocytoma

## Abstract

**Background:**

Pheochromocytoma is a neuroendocrine tumor, and its treatment is dependent on surgical resection. Due to the wide availability of cross-sectional imaging, pheochromocytomas are commonly seen as small tumors less than 10 cm in size and are mostly treated with minimally invasive surgery. Their concomitant presence with horseshoe kidney or other anatomical and vascular anomalies is rare. Herein, we present a surgically complex giant pheochromocytoma case who underwent an open left radical adrenalectomy.

**Case presentation:**

A 41-year-old Hispanic female presented with a 12 × 8 cm left hypervascular adrenal mass, pelvic horseshoe kidney, and severely dilated large left retro-aortic renal vein which branched into a left adrenal vein, congested left ovarian vein, and left uterine plexus. She was managed with insulin and metformin for uncontrolled diabetes with an A1c level of 9% and doxazosin for persistent hypertension. Clinical diagnosis of pheochromocytoma was confirmed with elevated urine and serum metanephrine and normetanephrine. The pre-operative ACTH was within normal range with a normal dexamethasone suppression test and 24-hour urine free cortisol. The adrenalectomy of the highly aggressive adrenal mass was performed via open approach to obtain adequate surgical exposure. Due to the large size of the tumor and its significant involvement with multiple adjacent structures, coordination with multiple surgical teams and close hemodynamic monitoring by anesthesiology was required for successful patient outcomes including preservation of blood supply to the pelvic horseshoe kidney. The histopathological diagnosis was pheochromocytoma with negative surgical margins. The patient was followed at 1, 4, 12, and 24 weeks postoperatively. She had a normal postoperative eGFR and was able to discontinue antihypertensive and antidiabetic medications at four weeks. She had transient adrenal insufficiency, which resolved at five months. The horseshoe kidney was intact except for a minimal area of hypo-enhancement in the left superior renal moiety due to infarction, which was significantly improved at six months.

**Conclusion:**

Our patient had a giant pheochromocytoma with anatomical variations complicating an already surgically challenging procedure. Nonetheless, with multiple provider collaboration, detailed pre-operative surgical planning, and meticulous perioperative monitoring, radical resection of the giant pheochromocytoma was safe and feasible with successful postoperative outcomes.

## Background

Pheochromocytomas are neuroendocrine tumors originating in the adrenal medulla. The tumors may be sporadic, though many carry mutations which have been inherited in an autosomal dominant pattern, and the latter is frequently found with other genitourinary anomalies and associated with increased risk of neuroendocrine renal tumors [1]. Symptoms of a pheochromocytoma arise due to the overproduction of catecholamines and, rarely, can be fatal. Surgical resection offers definitive treatment for a pheochromocytoma; however, patients are at significant risk for major adverse cardiac complications in the perioperative period. Concurrent presence of variant anatomy may further complicate the surgery. Horseshoe kidney with giant pheochromocytoma has not yet been described in the literature, to our knowledge. Therefore, we report a case of a sporadic, complex pheochromocytoma demonstrated by a large hypervascular adrenal mass, pelvic horseshoe kidney, and retro-aortic renal vein that underwent open left adrenalectomy.

## Case presentation

A 41 year-old Hispanic female with known uncomplicated type 2 diabetes mellitus for about 8 years treated with metformin presented to the emergency department with complaints of chest and abdominal pain, dyspnea, intractable nausea, and vomiting. She was in acute distress with tachycardia and hypertension, found to be in diabetic ketoacidosis, and diagnosed with an active COVID-19 infection prompting hospitalization. She was admitted to ICU and treated with continuous insulin infusion and IV fluids. She also acknowledged longstanding complaints of headaches, diaphoresis, and palpitations started several years ago prior to this presentation. Given chest pain at initial presentation, and elevated serum troponin level, she underwent further evaluation with CT chest, which revealed a 12 × 8 cm adrenal mass and a horseshoe kidney. The clinical diagnosis of pheochromocytoma was confirmed with elevated urine and serum metanephrine and noremetanephrine. Her diabetic ketoacidosis improved, and her COVID-19 infection was asymptomatic during her admission. She was discharged with long-acting insulin, and an alpha-blocker (terazosin), and the initial treatment plan was to proceed with resection of her adrenal mass. Nonetheless, her planned surgery was cancelled a few times due to patient’s insurance problems, and incompliance with use of phenoxybenzamine.

**Fig. 1:**
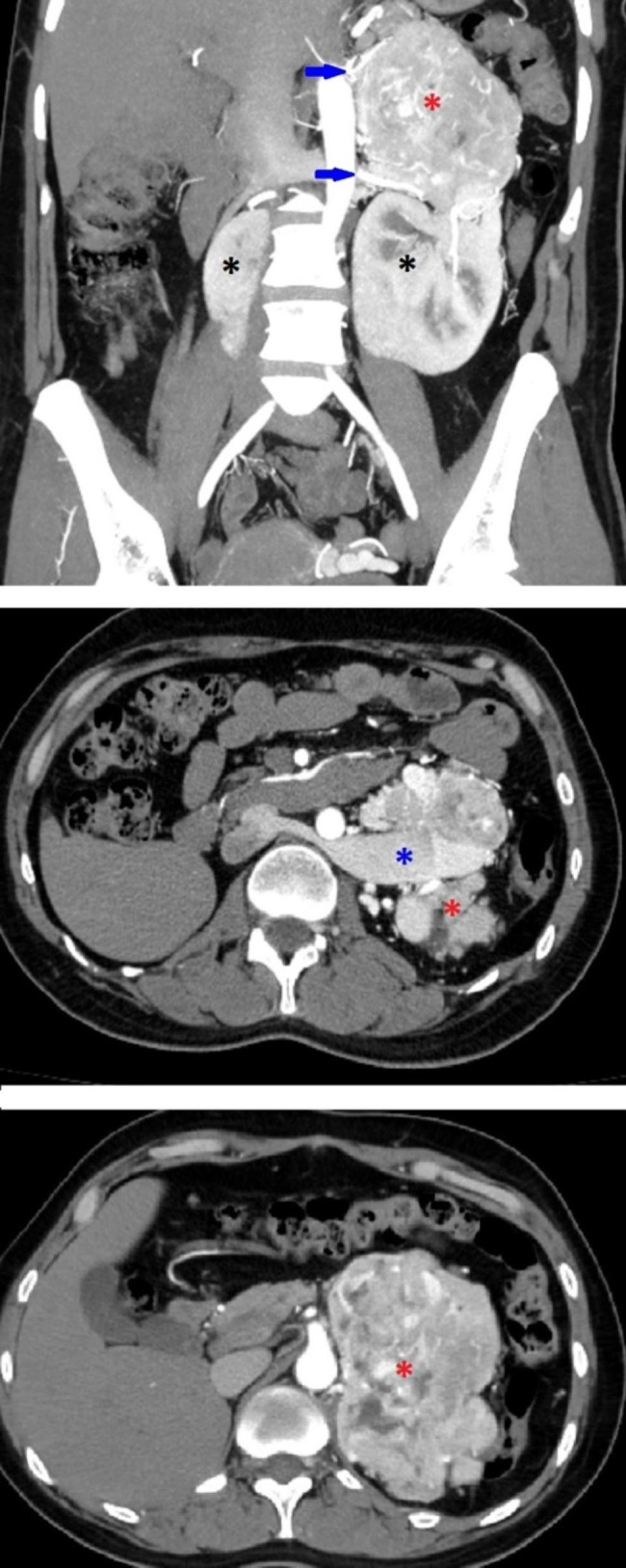
The preoperative CT angiogram in the coronal (**A.**) and axial (**B.**, **C.**) planes shows a large left adrenal mass (red asterisk), pelvic horseshoe kidney (black asterisks), severely dilated retro-aortic renal vein (blue asterisk) and several large aortic branches supplying the giant adrenal mass (blue arrows)

**Fig. 2:**
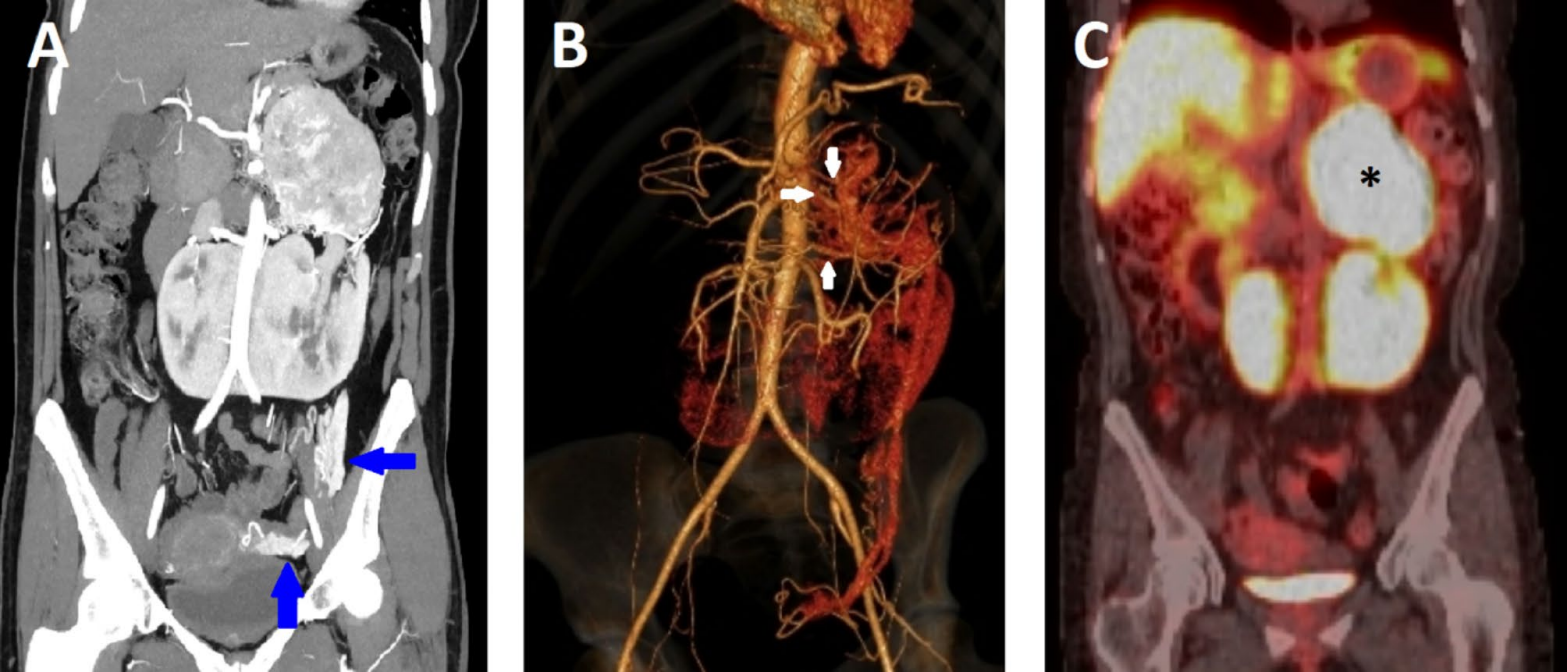
(**A.**) The preoperative CT angiogram, Maximum Intensity Projection (MIP), in the coronal plane shows a dilated left ovarian vein and uterine plexus (blue arrows) suggesting congestion caused by blockage of retro-aortic left renal vein outflow due to the large adrenal mass and aortic compression. (**B.**) The 3D vascular reconstruction demonstrating arterial vascular supply of the mass (white arrows). (**C.**) PET/CT Copper Cu 64 Dotatate demonstrating the large intensely Dotatate avid mass (black asterisk) superior to the left moiety of the patient’s horseshoe kidney and no evidence of Dotatate avid metastatic disease

About 22 months after initial diagnosis of pheochromocytoma, the patient was ready for surgery. The pre-operative assessment revealed normal levels of ACTH with a normal dexamethasone suppression test and 24-hour urine free cortisol. The serum renin, aldosterone, and DHEAS were in the normal range which excluded co-secretion from the pheochromocytoma. Further workup with CT chest abdomen pelvis and PET/CT Copper Cu 64 Dotatate scans were negative for metastasis and additional paragangliomas. CT angiogram for surgical planning revealed the large hypervascular adrenal mass, pelvic horseshoe kidney, severely dilated left retro-aortic renal vein which branching into an enlarged left adrenal vein, congested left ovarian vein, congested uterine plexus, and a minimum of three prominent arteries supplying the mass branching from the abdominal aorta, left renal artery, and L1 lumbar artery (Fig. 1 and Fig. 2). The mass appeared to be highly aggressive due to worrisome features on imaging, which include the large size of the mass, hypervascularity, and central areas of necrosis. There was loss of a clear fat plane between the mass and the pancreas which was concerning for invasion as well. Pre-operatively, she was treated with 14 days of phenoxybenzamine and a high salt and water diet. At this time, she was also on insulin and metformin for uncontrolled diabetes with an A1c level of 9% and doxazosin for persistent hypertension.

On the day of surgery, the patient confirmed that she had taken her morning dose of phenoxybenzamine. A radial arterial line was placed prior to the induction of general anesthesia. After the uneventful induction of general endotracheal anesthesia, a large bore central line was placed in the right internal jugular vein. A Belmont rapid infusion pump used for blood loss replacement was primed and connected to one lumen of the central line while a multi-channel infusion pump used for administering vasoactive medications was connected via a manifold to the second lumen of the central line. The medications chosen for the multi-channel infusion pump were nicardipine, remifentanil, vasopressin and norepinephrine. A 7 Fr rapid infusion catheter was also placed in the patient’s right antecubital fossa to provide redundancy for high-volume intravascular resuscitation.

**Fig. 3:**
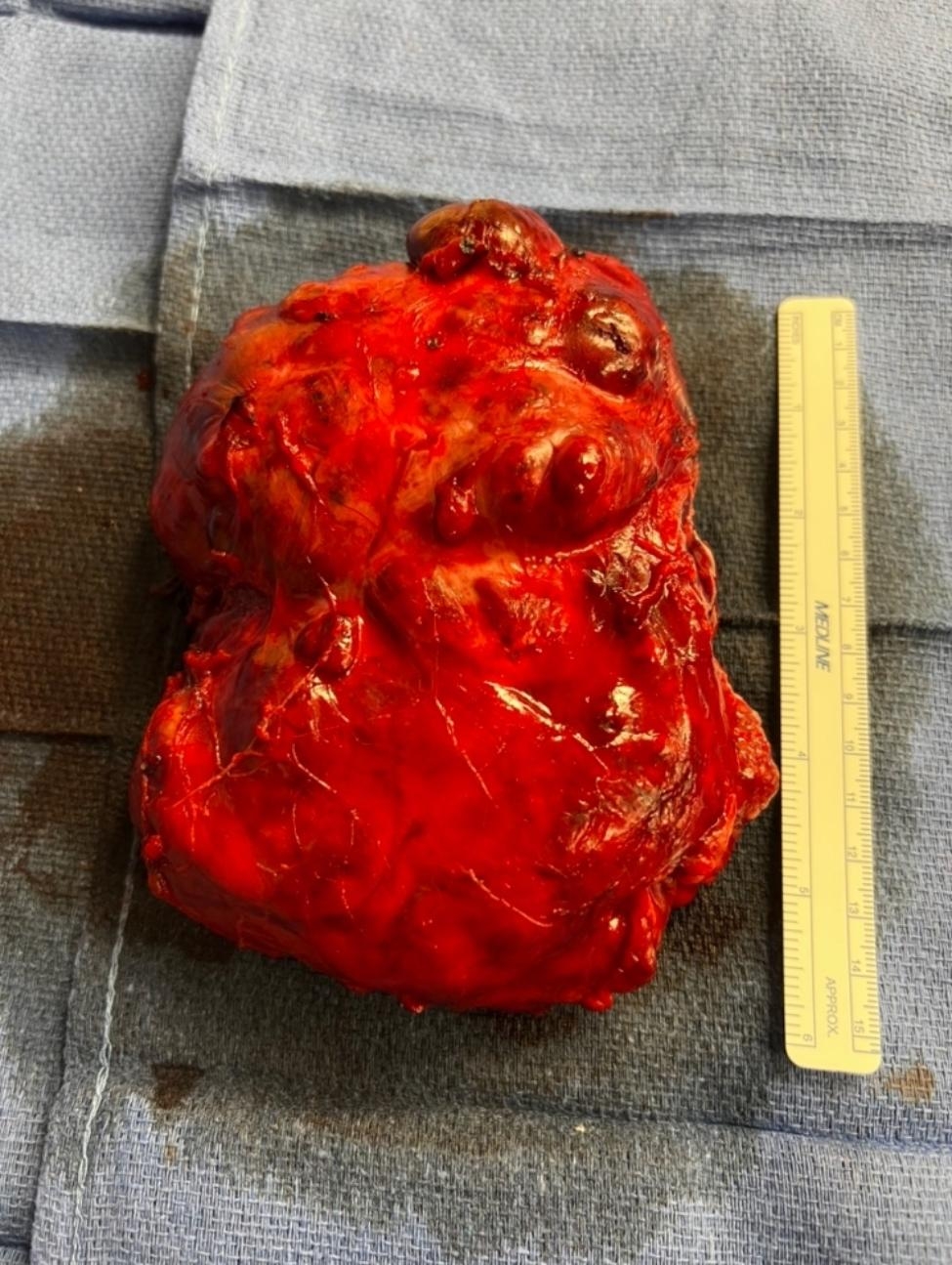
The en-bloc resected giant pheochromocytoma with overlying intact capsule, gross image

**Fig. 4:**
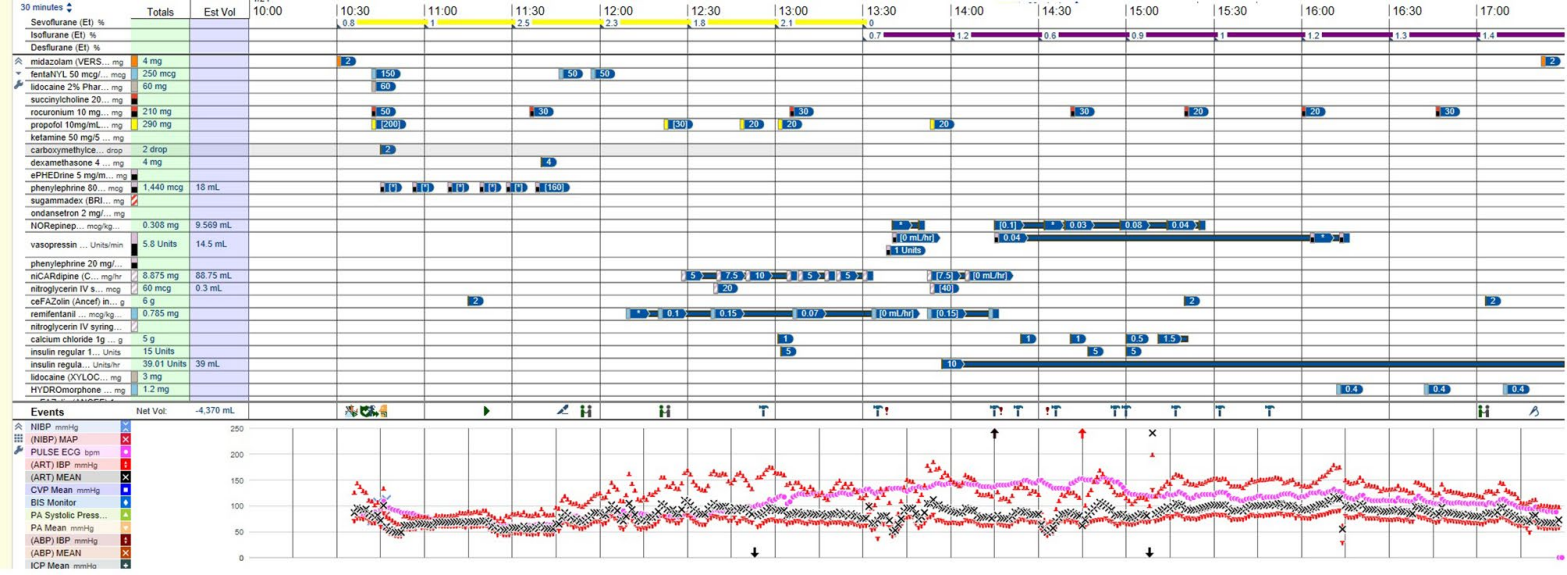
The anesthesia record with overview of the entire intraoperative course in relation to the patient’s hemodynamics and changing requirements in vasoactive infusions

A large midline laparotomy incision was performed for the surgical procedure. Intra-operatively, the patient’s systolic blood pressure was sensitive to palpation and manipulation of the mass, fluctuating between 60 and 190 mmHg, though remaining at an overall acceptable level throughout the procedure. The large adrenal mass was compressing the spleen, tail of the pancreas, splenic vessels, and aorta without significant tumor invasion. The tumor directly invaded the distal one third of the mesentery of the transverse colon, anteriorly, and the left crus of the diaphragm, posteriorly. To obtain vascular control and avoid excessive catecholamine discharge into the systemic circulation, the left adrenal vein, which was the tributary of the left renal vein, and direct venous branches of the inferior vena cava, which drained the adrenal mass, were first transected, causing engorgement of the mass and diffuse oozing of blood from its surface. It was not considered feasible to attempt in decreasing blood loss by performing initial transection of the arterial branches prior to transecting the vein, as the arteries were located posterior and deep to the adrenal mass. This would have also caused significant influx of catecholamines into the systemic blood circulation through the intact adrenal vein since continuous traction of the mass was needed for dissection. Due to significant compression of the spleen and splenic vessels by the mass posterosuperiorly and difficult dissection in the distorted surgical anatomic plane, an unavoidable 3 cm splenic laceration occurred, requiring splenectomy. The left superolateral portion of the mass invading into the left crus of the diaphragm was also resected, and the diaphragm was repaired with primary suture closure. Colorectal surgery assisted in exploring and repairing an approximately 8 cm mesenteric defect. Vascular surgery assisted in dissection of the adrenal mass medially off the aorta and in suture ligation of two large arteries branching from aorta. The significant compression of the aorta by the large adrenal mass required a sharp and meticulous dissection to avoid positive surgical margins and aortic injury. The mass was then resected en bloc with an intact overlying capsule (Fig. 3). The case, otherwise, was completed without additional major complications, despite abnormal anatomy, labile blood pressure, and dilutional coagulopathy as a result of massive transfusion. Particularly, three distinct epochs were noted during the entire case in terms of patient’s hemodynamics and the changing requirements in vasoactive infusions as the case progressed (Fig. 4). The first epoch was from the induction of general anesthesia (a little after 10:30 am) until the start of actual surgical dissection (around 11:45 am). With minimal stimulus, this period was characterized by the need for frequent small bolus doses of phenylephrine to support the blood pressure as well as relatively low levels of anesthetic gas administration. The second epoch of time was from the start of actual surgery (around 11:45) until the transection of the adrenal vessels (around 14:13 pm). This second period required infusions of nicardipine and remifentanil to blunt the hypertension and tachycardia spikes that were occurring due to tumor manipulation. The specific agents of nicardipine and remifentanil were chosen due to their easy titratability characteristics as well as their swift and reliable offset of action times. The third and final epoch was from the transection of the adrenal vessels (around 14:13 pm) until the end of surgery. At the start of this third period the nicardipine and remifentanil infusions were abruptly stopped and instead the new tendency towards hypotension (due to the new absence of catecholamine secretion) was offset by norepinephrine and vasopressin infusions. These norepinephrine and vasopressin infusions were eventually weaned and discontinued prior to the end of the case. Due to the large size of the tumor and its significant involvement with multiple adjacent structures, intra-operative blood loss was large with acute bleeding episodes. Blood loss was continuously monitored to quickly replace product through central venous access via Belmont rapid blood infuser. Total blood products transfused for the case were 20 units of packed red blood cells, 20 units of fresh frozen plasma, and three units of apheresis platelets. Serial arterial blood gases were drawn hourly throughout the case and serum glucose and electrolyte disturbances were corrected as needed. Although vasoactive infusions had been discontinued by the end of the case, due to the massive fluid shifts that occurred in her complex case, it was determined safest to keep the patient intubated and sedated overnight, should any further hemodynamic or metabolic derangements materialize. She was safely extubated on the morning of post-operative day one without complication.

**Fig. 5:**
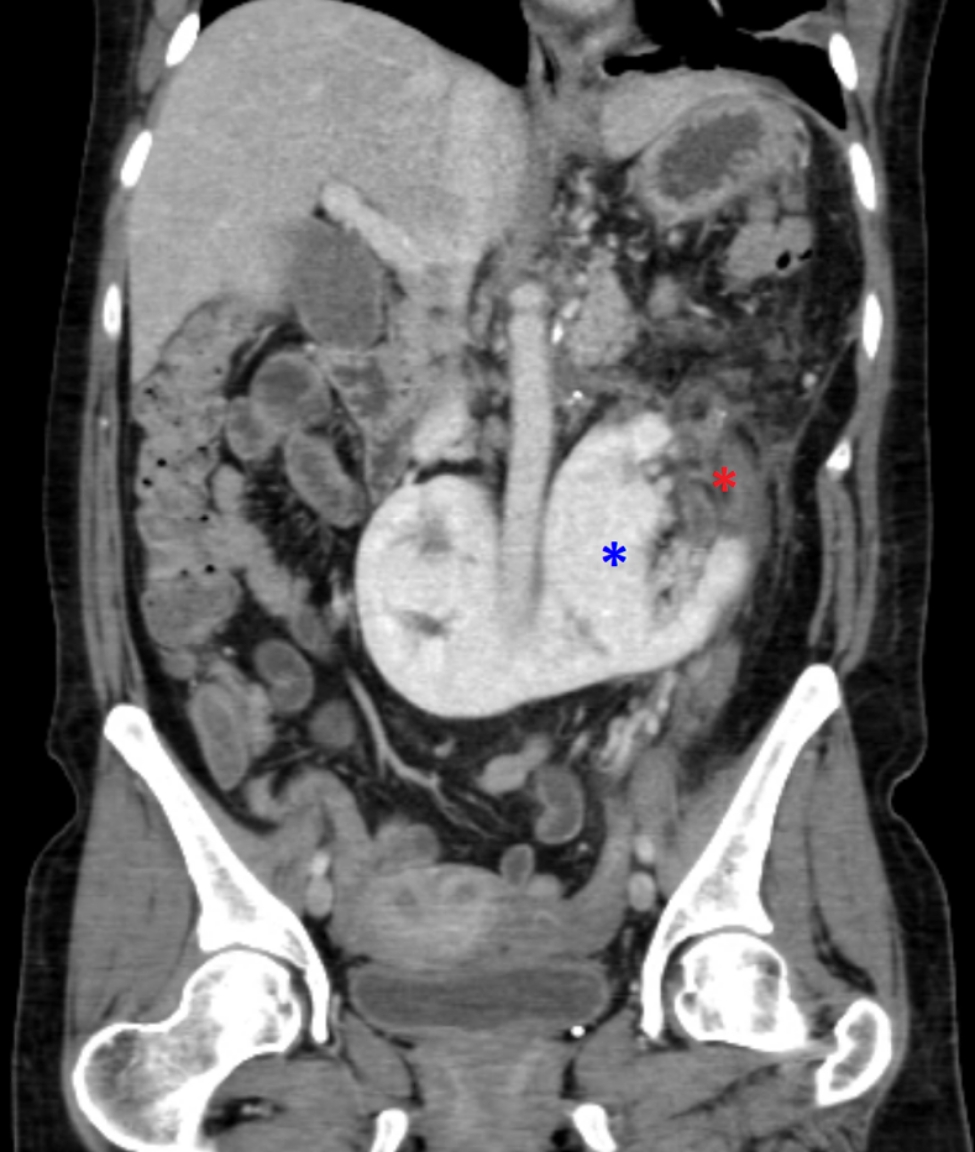
(**A:**) The postoperative one-week contrasted CT chest/abdomen/pelvis in coronal plane demonstrating preserved pelvic horse-shoe kidney (blue asterisks) with area of hypoenhancement (red asteriks)

The patient received her post-splenectomy vaccinations during her hospitalization. On post-operative day three, ACTH was elevated to 1073 pg/mL. Her AM cortisol was in the normal range at 10.4 mcg/dL. A Cosyntropin stimulation test showed peak cortisol of 10.8 mcg/dL (a normal response is peak cortisol of 18 mcg/dL or higher), diagnostic of adrenal insufficiency. The adrenal insufficiency was asymptomatic and required no treatment. Her blood glucose normalized, and insulin was discontinued. The patient was discharged on postoperative day six. Postoperative CT scan confirmed that the horseshoe kidney was preserved with some hypo-enhancement due to infarction in the left superior renal moiety (Fig. 5). This infarction likely occurred after transection of the left adrenal vein and the few veins branching from the inferior vena cava, which may have drained the left superior renal moiety in addition to the giant adrenal mass due to the atypical anatomical variants of blood supply to the horseshoe kidney.

**Fig. 6:**
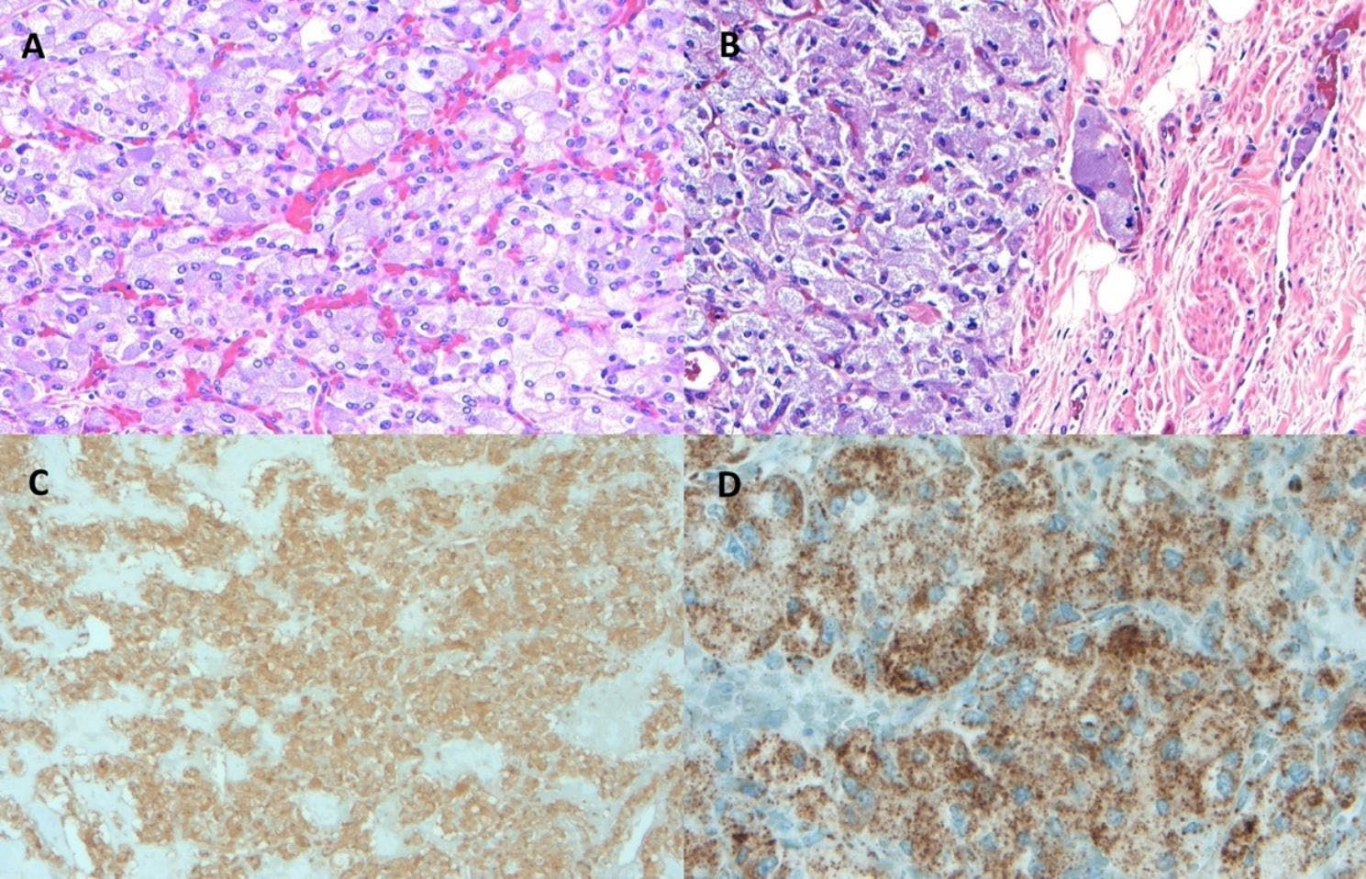
(**A:**) 10X, H&E. Tumor cells growing in nests called zell-ballen nests. (**B:**) 10X, H&E. Lympho-vascular invasion. (**C:**) 10X, Synaptophysin stain, positive. (**D:**) 10X, SDHB stain, positive

**Fig. 7:**
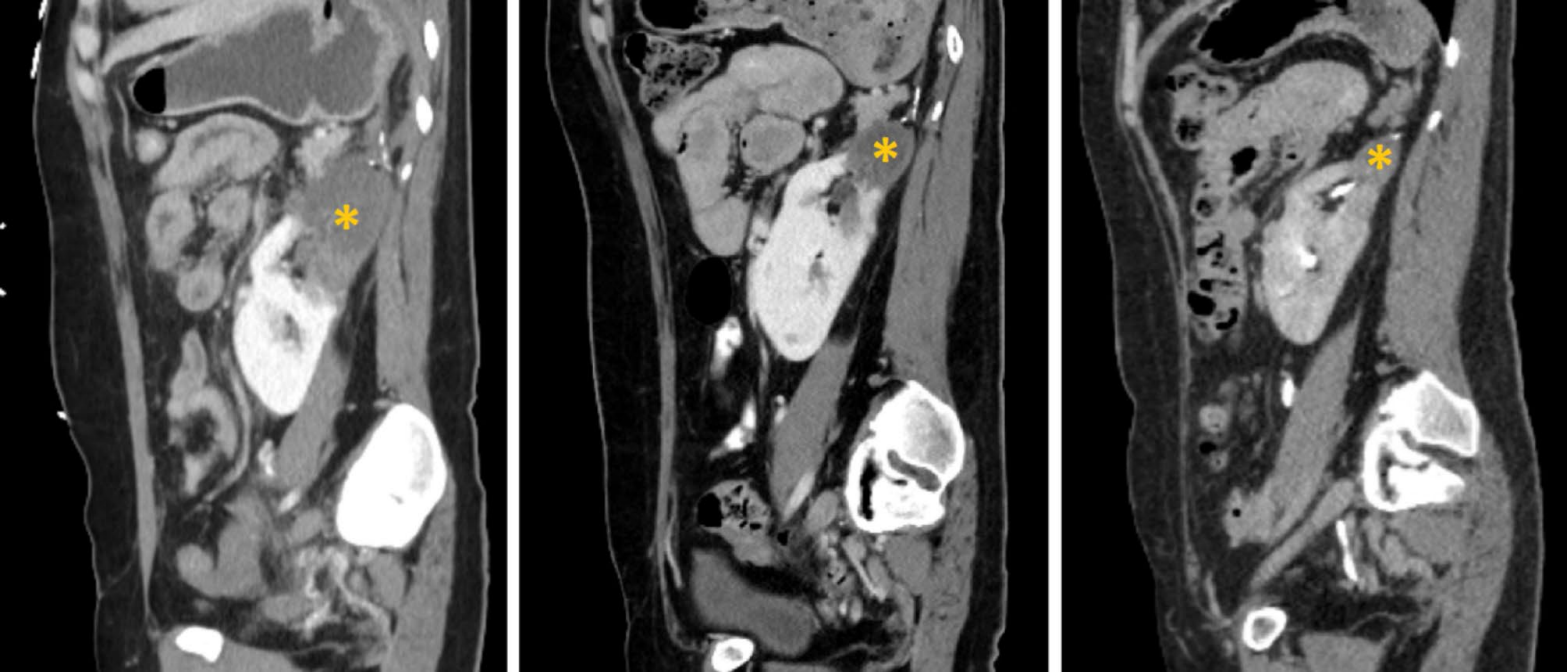
The postoperative contrasted CT chest/abdomen/pelvis in the sagittal plane demonstrating horseshoe kidney with improving hypoenhancing area in the left superior renal pole moiety (yellow asterisk), measuring 7.8 × 4.5 cm, 4.2 × 3.2 cm, and 2.1 × 1.7 cm at postoperative weeks of 1, 12 and 24, respectively

The histopathological examination revealed a pheochromocytoma, 12.6 cm in greatest dimension, with lymphovascular and capsular invasion (Fig. 6). Chromogranin and synaptophysin were diffusely positive. The surgical margins were negative. Immunohistochemistry for tumor suppressor, Succinate dehydrogenase B subunit (SDHB) mutation was negative. The patient underwent genetic testing of 84 genes associated with hereditary cancers, including VHL, SDHB, multiple endocrine neoplasia, and Neurofibromatosis type 1, and it was negative for any mutation, suggesting sporadic pheochromocytoma. One-month after surgery, she was able to discontinue antihypertensives and antidiabetics and her post-operative eGFR was > 90. Routine surveillance with CT examination at 3-months had demonstrated an intact pelvic horseshow kidney with the exception of a minimal hypo-enhancement in the left superior renal pole moiety and no recurrence of the pheochromocytoma. At 5 months post-operatively, the ACTH normalized at 47.5 pg/mL, and adrenal insufficiency resolved. Her HbA1c was normal at 6% off all anti-diabetes medications. At 6 months, the hypoenhancing area in the kidney significantly improved (Fig. 7).

## Discussion and conclusions

Originally derived from the neural crest, pheochromocytomas are neuroendocrine tumors of the adrenal medulla that arise from chromaffin cells. These tumors may be sporadic or hereditary, manifesting as part of a genetic syndrome such as Multiple Endocrine Neoplasia type 2, Von Hippel-Lindau disease, and Neurofibromatosis Type 1. The annual incidence of a pheochromocytoma is 2 to 9.1 per million adults and, when sporadic, commonly become apparent at 30–50 years old [2]. In our case, although only 84 common genes were evaluated, genetic evaluation revealed no mutation suggestive of hereditary pheochromocytoma. Moreover, unlike our case, hereditary pheochromocytomas are reported to occur at younger ages with smaller sized tumors, and, when compared to sporadic pheochromocytomas, are more often asymptomatic [3].

Although only estimated to occur in 24% of pheochromocytoma patients, the tumor’s secretion of catecholamines give rise to the classic triad of palpitations, diaphoresis, and headache [4]. Additional symptoms may include hypertension which can be refractory to treatment, flushing, abdominal pain, and hyperglycemia, as our patient demonstrated on initial presentation in diabetic ketoacidosis. In rare occasions, patients can present with “pheochromocytoma crisis” in cardiogenic shock, resulting in multiple organ failure. Furthermore, our patient had post-operative transient adrenal insufficiency. Adrenal insufficiency can develop following unilateral adrenalectomy in cases of overt or subclinical Cushing’s syndrome because of suppression of cortisol production from the contralateral adrenal gland [5]. This patient did not have evidence of cortisol hyper-secretion preoperatively. The incidence of adrenal insufficiency followed unilateral adrenalectomy for pheochromocytoma has not been reported in the medical literature, but it is thought to be rare [6].

Adrenal tumors are commonly benign. Conversely, adrenocortical carcinoma is a rare malignant adrenal mass that produces steroids and androgens. It has also been described in few cases to mimic pheochromocytomas [7]. In addition, the reported prevalence of malignant pheochromocytomas, defined by metastasis into non-chromaffin cells, ranges between 5 and 26% [8]. Local invasion does not diagnose malignancy. Surgery plays a critical role in malignant pheochromocytoma, as a possible curative treatment by adrenalectomy. Genetic testing of the SDHB mutation as part of pathologic analysis after resection gives additional information regarding the patient’s predisposition to developing pheochromocytomas, or in the case of malignant masses, prognosis, since shorter survival has been associated with positive SDHB mutations [3]. In our case, there was no signs of metastasis in anatomical and functional imaging, and SDHB mutation was also negative.

Minimally invasive surgery with laparoscopic adrenalectomy is preferred for smaller pheochromocytomas due to lower rates of intraoperative hemodynamic instability, less intraoperative blood loss, and shorter postoperative hospital stays [9]. However, the 2014 Endocrine Society Clinical Practice Guideline recommends open adrenalectomy for pheochromocytomas > 6 cm given high probability of a malignant functional tumor. Furthermore, complex anatomy can negatively impact the success of laparoscopic surgery. In the presented case, the final greatest dimension of tumor was 12.6 cm and concomitant pelvic horseshoe kidney and retro-aortic left adrenal vein were present. To our knowledge, this is the first case reporting a giant pheochromocytoma (12 cm in size) with a simultaneous horseshoe kidney. Previously few case reports demonstrated their co-existence, albeit all pheochromocytomas treated were smaller, 3 and 5 cm respectively [10, 11]. Horseshoe kidneys are rare entities, found in approximately 0.25% of the population and have a male predominance. Renal vein anomalies have an incidence reported in up to 23% of patients with horseshoe kidneys [12], as was identified in our patient with a retro-aortic renal vein. Given the patient’s anatomic variations and very large tumor size, an open radical surgical resection with adequate exposure was of paramount importance.

The additional challenge of adrenalectomy for pheochromocytoma is perioperative hemodynamic instability in the form of both severe hypertension and hypotension. Hypertension is observed with manipulation or palpation of the pheochromocytoma. Conversely, extreme hypotension arises upon interruption of the vessels leading to a sudden decrease in catecholamine secretion. Hypotension is further exacerbated by decreased vascular volume by intraoperative blood loss. In a study of 67 patients who underwent surgery for pheochromocytoma, changes in blood pressure greater than 30% of the patient’s pre-operative mean arterial pressure were observed in 97% of patients, with 36% experiencing hypertension and 93% experiencing hypotension [13]. Five or more episodes of severe hypotension correlated with longer stays in the intensive care unit due to the increased risk of the patient’s developing end-organ failure [13]. Intra-operatively, the individual patient’s course remains unpredictable and requires close monitoring by the anesthesiologist. Few factors, including tumor size, selective alpha-blockade, and operative approach, have been associated with intraoperative hemodynamic stability [14].

Thorough pre-surgical planning and close coordination among urologist, anesthesiologist, endocrinologist and other surgical teams are essential to prevent severe perioperative complications. Moreover, Abdou et al. reported a challenging case of a 5.5 cm left adrenal mass and horseshoe kidney, which failed surgical resection twice previously. The first attempted laparoscopic surgery was aborted due to severe adhesions, whereas the second attempted open surgery was aborted due to severe bleeding [11]. Only after a preoperative coil embolization of three large feeding arteries arising from the left renal artery and supplying the left adrenal mass, they could resect the mass in the third attempted open surgical resection successfully, with significantly low blood loss, 250 ml. Although pre-operative embolization could have potentially reduced the blood loss in our case, embolization is not without risks as it can precipitate hypertensive crisis, thus its role in treatment of pheochromocytoma still remains controversial. Furthermore, although the 3 cm splenic laceration was quite small, due to massive fluid shifts during the case, control of the bleeding was difficult and hemostasis could not be achieved sufficiently, therefore we elected for a splenectomy. Although, current literature suggested that immunization might be effective even in the immediate perioperative time for postsplenectomy infection prophylaxis [15], given the proximity of the giant adrenal mass to the spleen and complexity of the anatomy in this presented case, a preoperative vaccination at least 2 weeks prior to the planned surgery could have been performed instead.

To our knowledge, this is the first case report describing a giant pheochromocytoma with a concurrent horseshoe kidney. She also had several additional anatomical variations, including the retro-aortic left renal vein, and congested left ovarian vein, complicating an already surgically challenging procedure. Wide resection of the highly aggressive adrenal tumor was required due to the symptomatic mass caused by catecholamine overproduction and the possibility of malignancy. The surgical principles of gentle retraction of the adrenal mass and early control of adrenal vein to prevent bursts of catecholamine release may not be easily feasible for complex large pheochromocytoma cases with anatomical anomalies. Nonetheless, with detailed anatomic studies, thorough pre-operative planning, multiple provider collaboration, and meticulous intraoperative monitoring, it is possible to minimize complications and achieve successful outcomes.

## Data Availability

Not applicable.

## List of abbreviations

ACTH: Adrenocorticotropin releasing hormone
CT: Computed tomography
PET: Positron emission tomography
SDHB: Succinate dehydrogenase B subunit
VHL: Von Hippel Lindau
